# Exploring presentation differences in multi-cultural youth seeking assistance for mental health problems

**DOI:** 10.1186/s40359-021-00571-0

**Published:** 2021-04-27

**Authors:** Stephane Shepherd, Cieran Harries, Benjamin Spivak, Anne-Sophie Pichler, Rosemary Purcell

**Affiliations:** 1 Centre for Forensic Behavioural Science, Swinburne University of Technology and Victorian Institute of Forensic Mental Health, 1/582 Heidelberg Rd, Alphington, VIC Australia; 2 The Centre for Youth Mental Health, The University of Melbourne, Melbourne, Australia

**Keywords:** Mental health, Youth mental health, Culturally and linguistically diverse Australians, Help-seeking behaviour

## Abstract

**Background:**

Mental ill-health can impact an individual’s capacity to interact with others, make decisions, and cope with social challenges. This is of particular importance for many Culturally and linguistically diverse (CALD) individuals who may be at various stages of the acculturation process. The increasing diversity of the Australian population necessitates informed and culturally relevant services that meet the needs of a changing demographic. However the extant research on the mental health needs of CALD Australians is limited. This study aimed to further our understanding of the mental health needs of young CALD Australians by exploring the mental health concerns and social factors exhibited by CALD individuals accessing community based youth mental health services in two major cities.

**Methods:**

We performed a series of logistic regression models to ascertain if a concert of factors (i.e., clinical, socio-economic, criminal justice system involvement, child maltreatment, social support) were associated with CALD status

**Results:**

Comparisons across factors revealed no significant differences between groups. A small number of correlates differentiated between CALD and non-CALD participants (mental illness diagnosis during childhood, family history of mental illness/suicide, sensation seeking, sensitivity to punishment, maternal overprotection) however these factors were no longer meaningful after adjustment for multiple comparisons.

**Conclusions:**

In help-seeking mainstream youth populations, cultural differences across clinical and environmental factors appear to be minimal.

The increasing diversity of the Australian population is well documented. This is most apparent in two of Australia’s largest cities, Melbourne and Sydney where more than 3.2 million overseas-born Australians reside [1]. Australians from non-English speaking backgrounds are often referred to as culturally and linguistically diverse (CALD) populations [2]. CALD communities are heterogeneous; they comprise people with diverse cultural norms, practices and traditions, languages, religions, family structures and life experiences [1–3]. Moreover, many individuals from CALD backgrounds have experienced distinctive socio-historical and pre- and post-migratory experiences and stressors which are often implicated in their sub-group’s collective health and wellbeing [4–7].

Mental health is one important marker, given its impact on one’s capacity to effectively interact with others, make decisions, relate to others and cope with social challenges. This is of particular importance for many CALD individuals who may be at various stages of the integration and/or acculturation processes, both of which can bestow unique challenges and personal stressors [3, 8, 9]. The extant research on the mental health of CALD Australians is limited [10]. A national survey from 2007 noted lower reported levels of life-time mental disorder for CALD Australians compared to the general population [11]. This appears to be especially pronounced for CALD individuals born in non-English speaking countries [11]. Other Australian studies have found similar, or marginally higher rates of mental ill-health in immigrant populations compared to majority culture populations [12, 13]. However analyses focusing on specific CALD sub-populations, particularly humanitarian arrivals, have uncovered high rates of selected mental health concerns [14–17]. Worldwide, refugees experience post-traumatic stress disorder at approximately ten times the rate of the general population [18]. A high occurrence of Post-Traumatic Stress Disorder has been identified among Australian refugee populations from South Sudan, Vietnam, Burma and Sri Lanka [17, 19–21]. Generally however, the available literature on the mental health of CALD populations has been small in scope and possesses definitional inconsistencies [10]. Accurately identifying CALD subjects in datasets (where country of birth, language spoken, and ethnicity are not consistently detailed) is often challenging. Furthermore, the term ‘CALD’ combines numerous heterogeneous cultural sub-groups, which when combined in aggregate form, precludes unique information about such sub-groups.

Progressing our knowledge on the mental health of CALD Australians is a constructive endeavour for several reasons. First, the increasing diversity of the Australian population necessitates informed and relevant services that can readily meet the needs of a changing demographic. Prior research has noted both low levels of mental health literacy and an underutilization of mental health services [22–26] for CALD residents in Australian settings. Cultural viewpoints on understanding or recognising mental ill-health (i.e., stigma, shame) may also prevent help-seeking and medical intervention [27–29]. Second, mental health practitioners may require an improved understanding of CALD interaction and presentation styles in order to enhance the effectiveness of the clinical encounter and therapeutic alliance. For example, CALD individuals may present with unique symptom reporting styles or exhibit culturally specific behaviours [30, 31]. Moreover, some CALD individuals may be unwilling to disclose personal health information or minimize psychopathology in clinical scenarios as a result of mistrust or a discomfort with impersonal, generic services [22, 32–34]. Third, an inability to address the mental health needs of CALD migrants can impact their ability to effectively integrate, engage with, and contribute to the broader society.

This study aims to further our understanding of the mental health of CALD Australians by exploring the mental health concerns and social factors exhibited by young CALD individuals accessing community based youth mental health services in the greater metropolitan regions of Melbourne and Sydney, Australia. In particular we aim to identify any differences in mental health problems (i.e., diagnostic information) and social factors (i.e., employment, family support, adverse life events, justice system involvement) between CALD and non-CALD participants. Findings will elucidate the key concerns faced by young CALD Australians who are seeking assistance for mental health problems.

## Method

### Sample

Established by Australia’s federal government in 2006, *headspace* provides physical and mental health services, drug and alcohol services, and vocational assistance to people aged 12–25 years with emerging and established mental health problems. Because *headspace* delivers a range of services informed by principles underpinning early intervention, service users may present with a range of psychosocial problems with varying degrees of severity, although mood and affective symptoms predominate [35]. Clinical services are delivered by general practitioners, psychologists, psychiatrists, and other allied health professionals, and are largely subsidised through publicly funded health-care schemes. Most young people either self-refer or are referred by family, friends, health professionals, or school counsellors.

All young people who attended one of four *headspace* services in Melbourne or Sydney, Australia, between January 2011 and August 2012, spoke English, and were capable of providing informed consent, were approached to seek their participation in a longitudinal cohort study examining the course of psychiatric disorders in this population [36]. Three of the centres are in outer-city suburbs characterised by socioeconomic disadvantage and limited private sector investment in mental health. The fourth centre is in a relatively affluent inner-city suburb. It should be noted, however, that since there are no defined geographical catchment areas for headspace centres, young people can attend a service irrespective of their place of residence. Prospective participants included those who were receiving clinical services at the time of the study as well as those who were waitlisted. Those who were significantly intellectually impaired (i.e., IQ < 65) and could not either provide informed consent or complete the assessment tasks were excluded from the study, while those who were acutely suicidal (as determined by their assessing or treating *headspace* clinician) were not approached to participate until their risk was reduced.

### Procedure

The human research ethics committees at the University of Melbourne and the University of Sydney approved the study. Following assessment by a *headspace* Access Team clinician or completion of their first treatment session, prospective participants were contacted by telephone or in person by research assistants (RA) with a minimum four-year graduate psychology degree to discuss the aims and nature of the study and to determine their interest in participating. Participants aged 15 years and older provided written informed consent, whereas those aged 12–14 years assented and written informed consent was provided by a parent or guardian. The RAs conducted semi-structured interviews with each participant using a range of clinical measures. Registered psychologists trained the RAs in the use of the measures, such that the RAs achieved very good inter-rater reliability on each measure (kappa ≥ 0.8) before recruitment commenced. Following the interview, the RAs provided participants with an iPad or laptop on which they completed several self-report measures. Participants received a $20 gift voucher to compensate the time associated with completing each assessment.

### Measures

#### Demographic and socioeconomic information

Participants’ age, sex, country of birth (and their parents’ country of birth), languages spoken, relationship status, accommodation status, living arrangements, education and employment status, financial difficulties, and social welfare entitlements were ascertained using questions adapted from the national census [37] and other published sources [38, 39].

#### Cultural and linguistic diversity

Researchers and practitioners alike often describe people as being culturally and linguistically diverse (CALD) if their primary language, cultural norms, and values differ from those of the mainstream community in which they reside [2]. The term is typically applied to those from non-English speaking backgrounds. Here, we have used the term to represent “people of colour”, a term which generally excludes White/Caucasian populations with largely European ancestry. As such, the CALD group in this study largely comprises people who were born (or whose parent[s] was born) in Africa, Asia, Pacific Islands, Latin America, the Caribbean, or the Middle East. This was to ensure that our CALD group was likely to be from a visibly non-White minority group in addition to possessing non-English speaking ancestry. Those who were born (or whose parents were born) in a primarily European-language-speaking country (including Australia), but reported primarily speaking a non-European language were also considered to be CALD. We considered people who identified (or whose parents identified) as Maori to be CALD. Although Maori people are Indigenous to New Zealand, the term Indigenous in the Australian context solely refers to those of Aboriginal and Torres Strait Islander ancestry. Aboriginal and Torres Strait Islanders are typically not included as CALD in the Australian context and as such were not included in the CALD group in this study. Because of the strong European heritage of both countries, those with Argentine or Uruguayan backgrounds were not considered to be CALD unless their primary language suggested otherwise (i.e., spoke a non-European language). Although Zulu is the language spoken most often in South Africa, English is the principal language of most South African migrants to Australia. Therefore, people with South African backgrounds whose self-reported primary language was either English or Afrikaans were not considered to be CALD. However, South African migrants who reported primarily speaking a non-European (and non-Afrikaans) language were considered to be CALD.

### Clinical measures

#### General psychopathology

The Kessler 10 (K-10) [40] was used as a broad measure of psychological distress. The scale comprises 10 questions that enquire about the respondent’s negative emotional states experienced during the past four weeks. The degree to which each item is experienced is measured on a five-point scale. Item scores are summed and range between 10 and 50. Scores between 25 and 29 suggest the presence of a moderately severe mental disorder, while scores of 30 and above are indicative of more severe psychopathology.

#### Anxiety

The generalized anxiety disorder scale (GAD-7) [41] is a seven-item self-report measure of the most salient diagnostic features of generalised anxiety disorder. The frequency with which each of the symptoms is experienced in the previous two weeks is rated on a four-point scale. Research has suggested that the GAD-7 is a valid screening tool for GAD in primary care settings and for assessing its severity in clinical practice and research [41]. Scale scores range from 0 to 21 with higher scores indicating more severe psychopathology. Scores of five, 10, and 15 indicate the presence of a “mild”, “moderate”, and “severe” anxiety disorder, respectively. The overall anxiety severity and impairment scale (OASIS) [42] consists of five items that assess the frequency and severity of anxiety, use of avoidance behaviours, and the extent to which anxiety interferes with the respondent’s social and occupational functioning. Items are scored on a five-point scale and represent the respondent’s self-reported experience over the past week.

#### Depression

The clinician-rated quick inventory of depressive symptomatology (QIDS-C_16_), [43] assesses the presence, during the previous seven days, of the major symptoms of depression as defined by the fourth edition of the diagnostic and statistical manual of mental disorders (DSM-IV). Its 16 items, reflecting depressed mood, sleep disturbance, appetite/weight disturbance, diminished interest, lowered energy/fatigue, poor concentration, self-criticism, and suicidal ideation are rated on a four-point scale and summed to provide a score ranging from 0 to 27. Scores above 16 are considered to indicate the presence of a severe depressive disorder.

#### Ruminative style

Rumination was assessed using a 10-item questionnaire [44] derived from a longer, validated scale [45]. Respondents indicated the extent to which they experienced each item on a four-point scale.

#### Mania

The young mania rating scale (YMRS) [46] measures the nature and severity of core manic symptoms experienced within the past 48 h. Ratings for the 11 items are based on the interviewee’s subjective report of his or her clinical condition. Additional information is based on the interviewer’s clinical observations. Seven items are scored on a four-point scale. The remaining four items are scored on an eight-point scale. These latter items are weighted more heavily to compensate for poor cooperation by those who are severely ill. Scores are summed and range from 0 to 60.

#### Psychosis

The risk of psychosis was assessed using the comprehensive assessment of the at-risk mental state (CAARMS) [47], a semi-structured interview designed for use by mental health professionals to assess the presence and severity of psychotic symptoms over the past 12 months. The schedule measures symptoms across several domains: positive symptoms, concentration and attention, emotional disturbance, negative symptoms, behavioural change, motor abnormalities, and general psychopathology. The Positive Symptom scale, used in this study, comprises four subscales: unusual thought content, non-bizarre ideas, perceptual abnormalities, and disorganised speech. These subscales were rated according to the intensity, frequency, and duration of the symptoms, their relationship to substance use, and associated level of distress. Pre-set thresholds on both the intensity and frequency of these symptoms were used to classify participants as “psychotic”, “at risk” for psychosis (based on their subthreshold psychotic symptoms), or “not at risk” for psychosis.

#### Personality

The behavioural inhibition/behavioural activation system (BIS/BAS) [48] was used as a broad measure of participants’ subjective personality style. It comprises 24 items that index the person’s behavioural inhibition, responsiveness to reward and punishment, drive, and fun-seeking. The items are rated on a four-point scale and summed.

#### Childhood-onset disorder

Participants’ recalled whether they had ever received a diagnosis of a mental (i.e., neurocognitive, emotional, or behavioural) disorder during childhood.

#### Substance use

The alcohol, smoking and substance involvement screening test (ASSIST) [49] is an eight-item self-report measure of tobacco, alcohol, and illicit drug use, and associated concerns. The responses to six of the items are summed and used to indicate the level of risk (i.e., “lower”, “moderate”, or “’high”) associated with each substance. Responses to the final item are not used in these calculations, but when endorsed, indicate the recency of injecting substance use (which may itself indicate elevated risk of substance-related harm).

#### Clinical stage

Each participant was assigned a clinical stage based on criteria established by McGorry et al. (2006) [50]. The clinical staging model comprises six discrete stages: stage 0 (asymptomatic people at risk of a disorder who have not yet presented for care); stage 1a (help-seekers with mild symptoms and functional impacts); stage 1b (people with attenuated syndromes, often with mixed or ambiguous symptomatology and moderate or severe functional impacts); stage 2 (people with discrete disorders [i.e., those presenting with clear psychotic, manic, or severe depressive episodes); stage 3 (people with a recurrent or persistent disorder); and stage 4 (people with a severe, persistent, and unremitting illness). Staging decisions are informed by clinical assessment of the person’s current symptomatology (severity, frequency, and type); characteristic mental features; age of onset and course of illness before presentation to health services; previous “worst ever” symptoms and treatment episodes (inc., hospital admissions); current level of risk of harm due to the person’s illness; previous suicide attempts or other risky behaviours; and current (compared to premorbid) levels of social and occupational functioning.

#### Clinical severity

Rated on a seven-point scale, the single-item clinical global impressions scale (CGI) [51] indicates the severity of the person’s illness with higher scores indicating a more severe presentation. The item is completed by the interviewer and represents his or her clinical impression of the interviewee’s presentation and level of functioning, derived from all available information obtained during the assessment.

#### Occupational functioning and disability

The social and occupational functioning scale (SOFAS) [52] is an observer-rated scale that provides a global assessment of one’s social and occupational functioning. Scores range from 0 to 100. Higher scores indicate a superior level of functioning. For the purpose of this study, scores were calculated based on the participants’ lowest level of functioning in the past year. The assessment of the person’s level of functioning is independent of the severity of his or her symptomatology and includes impairments that are caused by either physical or mental disorders. To be considered, impairments must be caused by the illness per se rather than a reflection of a lack of opportunity or environmental limitations. The 12-item version of the World Health Organization Disability Assessment Schedule (WHODAS 2.0) [53] was used to examine participants’ difficulties in performing daily life activities during the past 30 days. Items are rated on a five-point scale and reflect six domains of functioning: cognition, mobility, self-care, social interactions, life activities (e.g., domestic responsibilities, leisure, work, and school), and participation in community activities. Total scores represent the simple sum of the 12 items and range from 0 to 48. Scores can also be scaled with a range from 0 to 100. Higher scores indicate a greater degree of disability.

#### Quality of life

Participants’ perceptions of their overall quality of life in the preceding four weeks were assessed using a single Likert item derived from the World Health Organization Quality of Life (WHOQOL) [54].

#### Family history of mental disorder

Participants were asked whether, to the best of their knowledge, any immediate family members (i.e., parents or siblings) had (1) ever experienced a serious psychological or emotional problem or (2) died by suicide [55]. Those who had no knowledge of their biological family did not complete this measure.

#### Forensic history

Participants were asked whether they had ever been (1) charged with a criminal offence; (2) convicted of a criminal offence; or (3) a victim of crime. Affirmative responses were followed up to clarify the nature of the crime and the outcome of any charges or convictions. Violence—whether perpetrated or experienced—was defined as any intentional behaviour involving threatened or actual physical harm (e.g., sexual or non-sexual assault, threats to kill or inflict injury). All other criminal behaviour (including theft, drug use or possession, property damage) was considered non-violent.

#### Child maltreatment

The childhood trauma questionnaire (CTQ) [56] was administered to assess participants’ experience of abuse and neglect during childhood and adolescence. Its 28 items measure the experience of three forms of abuse (physical, sexual, and emotional) and two forms of neglect (physical and emotional); a three-item scale is used to detect false-negative reports. Respondents rate the extent to which they experienced each item on a five-point scale. Higher scores indicate more frequent maltreatment; defined thresholds are used to classify cases according to their severity (i.e., “none”, “low”, “moderate”, or “severe”).

#### Parental style

A short version of the parental bonding instrument (PBI) [57] was used to assess participants’ recollection of their parents’ behaviour toward them. The scale comprises 18 items, which measure both maternal and paternal care, overprotection, and authoritarianism. Respondents rate the frequency with which they experienced each item during childhood on a four-point scale and the scores are summed.

#### Social support

A 20-item questionnaire [58] was used to assess the quality of participants’ interactions with their family, friends, and partner (where applicable). The items are scored on a four-point scale and summed to yield six indexes that measure either positive or negative qualities within these domains. The composite scores are scaled such that they theoretically range from 0 to 1 with higher scores reflecting greater endorsement of the underlying items.

#### Stigmatisation

Three items were adapted from the discrimination scale in the quality of life in newly diagnosed epilepsy instrument (NEWQOL) [59]. Participants were asked whether or not, because of their mental health problems, others: (1) are uncomfortable with them; (2) treat them as inferior; or (3) prefer to avoid them.

### Statistical analysis

All analyses were conducted using R version 4.0.2 [60]. For all standard analyses, two-tailed tests were used with a significance level of *α* < 0.05. We corrected for multiple comparisons using a false discovery rate (FDR) of 5% [61]; *q*-values were calculated using the *qvalue* package [62].

#### Missing data

Of the 1615 eligible help-seekers approached by the RAs to participate, 806 consented (of whom four subsequently withdrew), representing a participation rate of 49.9%. The majority of those records retained for analysis (662 [82.5%]) were incomplete. Nearly three-quarters (483 [73.0%]) of these records were missing ≤ 5.3% of their values (range, 0.9–84.7%). Only three (2.7%) variables were complete. The number of missing values across variables ranged from one (0.1%) to 319 (39.8%).

Missing indicators were created for each variable with missing data. Large correlations among variables with missing values suggested that the propensity for missingness in a given variable was related to the presence of missing values in other variables. Fitted binary logistic regression models with the indicator variables as outcomes demonstrated that missingness was associated with observed values, suggesting they were at least partly *missing at random* [63].

We estimated missing values using multiple imputation by chained equations (MICE) [64, 65], a technique that involves imputing each missing value with multiple values estimated from the posterior predictive distribution of the missing data conditional on the observed data. Variables were imputed using flexible additive regression models as implemented in the *aregImpute* function from the *Hmisc* package [66]. *aregImpute* accounts for all aspects of uncertainty in the imputations by using the bootstrap to approximate the process of drawing predicted values from a full Bayesian predictive distribution. We generated eighty complete datasets. Trace plots demonstrated that the results were stable across the iterations for each imputation, suggesting convergence had been achieved. Density plots showed the distributions of the imputed and complete data were similar.

#### Descriptive analyses

Numeric variables were expressed as an arithmetic mean and standard deviation. Categorical variables were expressed as counts and percentages. The distributions of characteristics of CALD and non-CALD help-seekers were compared using independent-sample *t*-tests and Pearson chi-square tests of independence. Mean differences (MD) and odds ratios (OR), and their associated 95% confidence intervals, were reported as the primary measures of effect.

#### Correlates of CALD status

Sixty-six variables of interest were classified to one of nine domains: demographic, financial security, mental disorder, substance use, functional impairment, forensic history, child maltreatment, social support, and stigma. Given the large number of variables, we performed a series of principal components analyses (PCA) using the *psych* package [67] to reduce the dimensionality of the dataset and eliminate redundancy among the covariates. We used the Kaiser–Meyer–Olkin (KMO) measure of sampling adequacy [68] to determine the proportion of variance in each set of variables that might be explained by underlying factors and Bartlett’s test of sphericity [69] to assess the suitability of the data for PCA. Principal components with eigenvalues greater than 1 were retained. Oblique (promax) rotation was performed on the variables to improve interpretability (see supplementary information). Component scores were computed for each participant and retained for subsequent analyses.

We constructed several logistic regression models using the *lrm* function in the *rms* package [70] to identify those factors that were associated with CALD status. Participants were categorised into CALD and non-CALD status as delineated earlier. We performed univariate analyses to examine the association of CALD status and each candidate variable. Then, we examined the association of CALD status and each of the eight variable domains by developing a series of restricted multivariate models comprising only those variables classified to each domain. We assessed model fit using a likelihood ratio test comparing each model to the null model and assessed model performance using the Nagelkerke *R*^2^ [71]. Calibration was determined by computing the mean squared deviation of each predicted probability from the true observed value of the outcome (i.e., the Brier score) [72]. The Brier score can be considered a weighted loss function in which increasing distance between predicted and observed values is penalised by a quadratic measure. It is a proper score function that ranges from 0 to 1. Although its numerical value has no direct meaning, lower scores indicate better performance. Internal validation was performed using bootstrap resampling (5000 replicates). Finally, we quantified the importance of each domain by comparing the fit of the restricted models to those of a fully adjusted multivariate model, in which all variables were entered simultaneously, using a likelihood ratio test.

#### Sensitivity analyses

Following multiple imputation (MI), each complete dataset is typically analysed separately using standard methods. Then, the *M* parameter estimates and their associated variances are pooled using Rubin’s rules to provide a single parameter estimate that incorporates both between- and within-imputation variability, thereby enabling correct inference [73]. However, problems arise when using MI in the context of PCA. Because of the variability in the imputed values, there is no guarantee that the eigenvector corresponding to a given eigenvalue is comparable across datasets. Consequently, pooling the eigenvectors (principal axes, factor loadings) using the order or the obtained eigenvalues of the covariance matrix estimated from each imputed dataset is likely to lead to misleading or meaningless results. Similarly, determining a common set of principal components across imputed datasets can be problematic, with the variability in the imputed values leading to different decisions being made for different datasets [74]. Considering these difficulties, we performed our analyses on a single dataset selected at random. To assess whether our results were sensitive to the variability in the imputed values, we conducted the same analyses in 10 additional datasets selected at random.

## Results

### Demographic characteristics

Participant characteristics are reported in Table 1. Most participants (718 [91.6%]) were born in Australia, thirty-five (4.9%) of whom identified as Australian Aboriginal or Torres Strait Islander. Of the 66 participants born overseas, 36 (54.5%) were born in Europe, New Zealand, Canada, or the USA. The remaining 30 participants were born in Asia (17 [25.8%]), Africa (10 [15.2%]), the Middle East (2 [3.0%]), and Latin America (1 [1.5%]). Similarly, most of the participants’ parents (558 [71.5%] of mothers and 520 [68.7%] of fathers) were born in Australia. Three in every five overseas-born parents (134 [66.0%] of mothers, 123 [56.7%] of fathers) were born in Europe, New Zealand, Canada, or the USA. Among those participants born in Australia, 283 (39.4%) had at least one parent who was born overseas; 93 (13.0%) had at least one parent who was born in Asia (51 [54.8%]), the Pacific Islands (16 [17.2%]), Latin America (11 [11.8%]), the Middle East (9 [9.7%]), and Africa (7 [7.5%]). Virtually all (771 [98.5%]) participants reported speaking English at home; 108 (13.8%) reported speaking more than one language. Nearly one in ten (63 [8.1%]) reported speaking a non-European language. Based on these characteristics, 128 participants (17.3% of the sample) were classified as CALD. Missing values precluded the classification of 62 participants.

**Table 1:** Baseline characteristics of CALD and non-CALD participants

	CALD [*n* = 128]	Not CALD [*n* = 612]	OR [95% CI]
*Demographic*			
Age [*range*, 12–25 years]	18.6 [3.2]^a^	18.2 [3.2]^a^	0.39 [− 0.22, 1.00]^b^
Female	88 [68.8%]	408 [66.7%]	1.10 [0.73, 1.66]
Australian-born	88 [68.8%]	587 [95.9%]	0.09 [0.05, 0.16]^gj^
*Education status*			
Not engaged in education	33 [26.0%]	202 [33.9%]	
School	39 [30.7%]	226 [37.9%]	1.06 [0.64, 1.74]
Vocational institution	16 [12.6%]	70 [11.7%]	1.40 [0.73, 2.70]
University	39 [30.7%]	98 [16.4%]	2.44 [1.44, 4.11]^gi^
*Employment status*			
Unemployed	70 [54.7%]	379 [61.9%]	
Full-time	9 [7.0%]	50 [8.2%]	0.97 [0.46, 2.07]
Part-time	49 [38.3%]	183 [29.9%]	1.45 [0.97, 2.17]
*Relationship status*			
Not in a relationship	84 [65.6%]	378 [61.8%]	
Married / living together (> 6 months)	4 [3.1%]	21 [3.4%]	0.86 [0.29, 2.56]
Non-spousal partner	40 [31.3%]	213 [34.8%]	0.85 [0.56, 1.28]
*Accommodation status*			
Boarding house / hostel	4 [3.3%]	10 [1.7%]	
Independent housing	116 [96.7%]	571 [98.3%]	0.51 [0.16, 1.65]
Welfare recipient	53 [52.0%]	221 [47.0%]	1.22 [0.79, 1.87]
*Mental disorder*			
Child-onset disorder	8 [7.1%]	100 [17.3%]	0.34 [0.16, 0.71]^fh^
*Clinical stage*			
Stage 0: Asymptomatic	3 [2.4%]	1 [0.2%]	
Stage 1a: Symptomatic	31 [24.4%]	235 [38.5%]	0.04 [< 0.01, 0.44]^gi^
Stage 1b: Attenuated syndrome	69 [54.3%]	312 [51.1%]	0.07 [< 0.01, 0.72]^fh^
Stages 2 +	24 [18.9%]	62 [10.2%]	0.13 [0.01, 1.30]^e^
Stage 2: Discrete disorder	21 [16.5%]	44 [7.2%]	
Stage 3: Recurrent or persistent disorder	3 [2.4%]	17 [2.8%]	
Stage 4: Severe, persistent, and unremitting illness	0 [0.0%]	1 [0.2%]	
*Psychosis*			
Not at risk	72 [57.1%]	347 [57.3%]	
At risk	45 [35.7%]	220 [36.3%]	0.99 [0.65, 1.48]
Met threshold for psychosis	9 [7.1%]	39 [6.4%]	1.11 [0.52, 2.40]
*Depression*			
None	28 [22.0%]	130 [21.3%]	
Mild	35 [27.6%]	204 [33.5%]	0.80 [0.46, 1.37]
Moderate	40 [31.5%]	172 [28.2%]	1.08 [0.63, 1.84]
Severe	23 [18.1%]	79 [13.0%]	1.35 [0.73, 2.51]
Very severe	1 [0.8%]	24 [3.9%]	0.19 [0.03, 1.49]
*Anxiety*^*c*^			
None	29 [23.2%]	152 [25.0%]	
Mild	35 [28.0%]	135 [22.2%]	1.36 [0.79, 2.34]
Moderate	29 [23.2%]	166 [27.3%]	0.92 [0.52, 1.60]
Severe	32 [25.6%]	154 [25.4%]	1.09 [0.63, 1.89]
Family history of mental illness or suicide	66 [53.2%]	381 [63.9%]	0.64 [0.43, 0.95]^e^
*Substance use*^d^			
Tobacco	76 [59.8%]	412 [67.3%]	0.72 [0.49, 1.07]
Alcohol	101 [79.5%]	531 [86.8%]	0.59 [0.36, 0.97]^e^
Cannabis	54 [42.5%]	324 [52.9%]	0.66 [0.45, 0.97]^e^
Cocaine	17 [13.4%]	94 [15.4%]	0.85 [0.49, 1.48]
Stimulants	27 [21.3%]	163 [26.7%]	0.74 [0.47, 1.18]
Inhalants	8 [6.3%]	57 [9.3%]	0.65 [0.30, 1.41]
Sedatives	17 [13.4%]	94 [15.4%]	0.85 [0.49, 1.48]
Hallucinogens	14 [11.0%]	122 [20.0%]	0.50 [0.28, 0.90]^e^
Opioids	6 [4.7%]	36 [5.9%]	0.79 [0.33, 1.92]
Other drug(s)	4 [3.1%]	23 [3.8%]	0.83 [0.28, 2.45]
Injecting drug use	2 [1.6%]	18 [3.0%]	0.53 [0.12, 2.32]
*Occupational functioning*			
Functional impairment	49 [38.6%]	227 [37.2%]	1.06 [0.72, 1.57]
*Quality of life*			
Poor	29 [23.6%]	159 [26.3%]	
Average	46 [37.4%]	221 [36.6%]	1.14 [0.69, 1.90]
Good	48 [39.0%]	224 [37.1%]	1.17 [0.71, 1.94]
*Forensic history*			
Non-violent charge	7 [5.6%]	43 [7.1%]	0.77 [0.34, 1.76]
Violent charge	3 [2.4%]	25 [4.2%]	0.57 [0.17, 1.92]
Violent victimization	20 [15.9%]	123 [20.2%]	0.75 [0.45, 1.25]
*Child maltreatment*			
Physical abuse	44 [34.9%]	187 [30.7%]	1.21 [0.81, 1.82]
Sexual abuse	25 [19.8%]	133 [21.8%]	0.89 [0.55, 1.43]
Emotional abuse	91 [72.2%]	411 [67.5%]	1.25 [0.82, 1.92]
Physical neglect	57 [45.2%]	279 [45.8%]	0.98 [0.66, 1.44]
Emotional neglect	83 [65.9%]	395 [64.9%]	1.05 [0.70, 1.57]
*Stigmatization*	37 [42.5%]	177 [45.5%]	0.89 [0.55, 1.42]
Values are counts and percentages. Percentages are expressed as a function of valid cases rather than total cases ^a^Values are means (standard deviations). ^b^Mean difference. OR = odds ratio; CI = confidence interval. ^c^Anxiety symptoms measured using the GAD-7. ^d^Lifetime use Significance testing: ^e^*p* < .05, ^f^*p* < .01, ^g^*p* < .001. Values corrected for multiple testing using a false discovery rate (FDR) of 5%: ^h^*q* < .05, ^i^*q* < .01, ^j^*q* < .001			

### Correlates of CALD status

Logistic regression was used to examine the extent to which CALD status was associated with a range of demographic, socioeconomic, clinical, and forensic indicators. The Box–Tidwell [75] procedure was used to test for linearity of continuous independent variables and their logits. Significance tests performed on the interaction between each continuous variable and its log were not significant (*p* > 0.05), suggesting that the data were consistent with a model in which the assumption was valid.

Table 2 reports the unadjusted and adjusted OR for the potential correlates of CALD status. Only two of the nine domain-based models were statistically significant (Table 3). Demographic characteristics and socioeconomic indicators were similar for both groups. Moreover, there were no differences in risk of substance use, levels of functional impairment, rates of criminal offending or victimisation, social support, or stigmatising experiences. The mental disorder model was statistically significant, *χ*^2^(13) = 31.25, *p* = 0.003, but explained only 2.3% of the variance in CALD status (bias-corrected Nagelkerke *R*^2^ = 0.023). CALD participants reported being less responsive to reward and punishment and reported experiencing lower drive and sensation-seeking tendencies than non-CALD participants. Moreover, they were less likely to report being diagnosed with a mental disorder during childhood and to report having an immediate family member (i.e., parent or sibling) who had experienced a mental disorder or died by suicide. None of these variables remained significant after correcting for multiple comparisons. The child maltreatment model was also significant, *χ*^2^(7) = 24.72, *p* < 0.001 (bias-corrected Nagelkerke *R*^2^ = 0.027). Although CALD participants reported higher levels of maternal overprotection than non-CALD participants, the observed difference was not significant following correction (Table 4).

**Table 2:** Correlates of CALD status

	OR_unadj_ [95% CI]	OR_adj_ [95% CI]	
		Restricted models	Full model
*Demographic*			
Age	1.05 [0.99, 1.11]	1.06 [> 1.00, 1.13]^a^	1.07 [0.98, 1.16]
Female	1.13 [0.75, 1.69]	1.12 [0.73, 1.68]	1.10 [0.64, 1.93]
Current relationship	1.19 [0.81, 1.78]	1.24 [0.83, 1.87]	1.12 [0.69, 1.78]
NEET	0.71 [0.38, 1.13]	0.63 [0.34, 1.04]	0.82 [0.40, 1.56]
*Accommodation status*			
Independent housing			
Boarding house / hostel	2.29 [0.55, 6.73]	2.14 [0.49, 6.82]	2.86 [0.48, 12.35]
*Financial security*			
Unable to meet basic need(s)	0.97 [0.74, 1.20]	0.97 [0.75, 1.20]	1.07 [0.72, 1.46]
Financial support required	0.94 [0.78, 1.14]	0.94 [0.78, 1.15]	0.88 [0.64, 1.26]
*Mental disorder*			
Clinical severity			
Normal, not ill at all			
Borderline mentally ill	0.43 [0.17, 1.35]	0.54 [0.21, 1.59]	0.68 [0.25, 2.44]
Mildly ill	0.52 [0.23, 1.48]	0.77 [0.33, 2.09]	0.91 [0.35, 2.92]
Moderately ill	0.65 [0.29, 1.88]	0.97 [0.41, 2.78]	1.15 [0.42, 3.82]
Markedly ill	0.67 [0.26, 2.07]	1.06 [0.38, 3.44]	1.34 [0.39, 5.10]
Severely ill	0.72 [0.12, 3.11]	1.49 [0.24, 6.86]	1.86 [0.22, 10.79]
Child-onset disorder	0.28 [0.11, 0.54]^a^	0.27 [0.11, 0.55]^a^	0.28 [0.11, 0.62]^a^
Mood and affective symptoms	1.01 [0.85, 1.21]	0.94 [0.74, 1.18]	0.78 [0.53, 1.16]
Behavioural activation	0.86 [0.71, 1.04]	0.81 [0.67, 0.99]^a^	0.82 [0.65, 1.04]
*Psychosis*			
Not at risk			
At risk	1.11 [0.74, 1.65]	1.09 [0.69, 1.74]	1.11 [0.66, 1.87]
Met threshold for psychosis	1.03 [0.45, 2.02]	1.11 [0.44, 2.37]	1.01 [0.38, 2.35]
Mania	0.98 [0.94, 1.02]	0.98 [0.93, 1.02]	0.98 [0.93, 1.04]
Familial mental illness or suicide	0.61 [0.42, 0.89]^a^	0.63 [0.43, 0.93]^a^	0.64 [0.41, 1.01]
*Functional impairment*	1.01 [0.84, 1.22]	1.01 [0.84, 1.22]	0.87 [0.57, 1.29]
*Substance use*			
Alcohol / cannabis / tobacco	0.93 [0.74, 1.13]	0.91 [0.71, 1.13]	0.90 [0.66, 1.16]
Stimulants and other drugs	1.09 [0.77, 1.21]	1.06 [0.81, 1.29]	1.04 [0.77, 1.34]
*Forensic History*			
Violent charge	0.59 [0.13, 1.47]	0.65 [0.12, 1.88]	0.60 [0.11, 2.31]
Non-violent charge	0.83 [0.36, 1.69]	1.02 [0.41, 2.24]	1.02 [0.34, 2.73]
Violent victimization	0.65 [0.37, 1.05]	0.68 [0.36, 1.17]	0.56 [0.26, 1.17]
Non-violent victimization	0.80 [0.41, 1.39]	0.97 [0.46, 1.77]	1.02 [0.44, 2.25]
*Child maltreatment*			
Child maltreatment	1.01 [1.00, 1.02]	1.00 [0.99, 1.02]	1.01 [0.99, 1.03]
Maternal care	0.97 [0.92, 1.02]	0.94 [0.88, 1.01]	0.96 [0.89, 1.05]
Maternal overprotection	1.20 [1.08, 1.33]^cd^	1.18 [1.04, 1.33]^b^	1.17 [> 1.00, 1.34]^a^
Maternal authoritarianism	1.13 [1.04, 1.22]^b^	1.09 [0.99, 1.19]	1.08 [0.97, 1.20]
Paternal care	1.00 [0.95, 1.05]	1.00 [0.94, 1.06]	0.99 [0.92, 1.07]
Paternal overprotection	1.08 [0.98, 1.18]	1.07 [0.94, 1.20]	1.09 [0.94, 1.26]
Paternal authoritarianism	1.00 [0.94, 1.07]	0.97 [0.90, 1.05]	0.95 [0.86, 1.05]
*Social support*			
Friend (positive interactions)	0.81 [0.40, 1.73]	0.78 [0.38, 1.68]	0.94 [0.34, 2.51]
Friend (negative interactions)	0.72 [0.31, 1.59]	0.70 [0.29, 1.52]	0.51 [0.19, 1.40]
*Stigmatization*	1.01 [0.82, 1.24]	1.01 [0.82, 1.24]	1.05 [0.76, 1.42]

Confidence intervals are bootstrapped (5000 replicates). NEET = Not in education, employment, or training. Stimulants and other drugs = cocaine and other stimulants, opioids, inhalants, hallucinogens, other (unspecified drugs), injecting drug use

Significance testing: ^a^*p* < .05, ^b^*p* < .01, ^c^*p* < .001. Values corrected for multiple testing using a false discovery rate (FDR) of 5%: ^d^*q* < .05

**Table 3:** Model fit and performance metrics for full and restricted domain-based models

	Likelihood ratio test	Index (original)	Index (bias-corrected)
*Demographic*	*χ*^2^(5) = 9.06, *p* = 0.11		
Nagelkerke *R*^2^		0.02	< 0.01
Brier score		0.14	0.14
*Financial security*	*χ*^2^(2) = 0.52, *p* = 0.77		
Nagelkerke *R*^2^		< 0.01	< 0.01
Brier score		0.14	0.14
*Mental disorder*	*χ*^2^(12) = 31.05, *p* = 0.002		
Nagelkerke *R*^2^		0.06	0.02
Brier score		0.13	0.14
*Substance use*	*χ*^2^(2) = 0.80, *p* = 0.67		
Nagelkerke *R*^2^		< 0.01	< 0.01
Brier score		0.14	0.14
*Functional impairment*	*χ*^2^(1) = 0.01, *p* = 0.92		
Nagelkerke *R*^2^		< 0.01	< 0.01
Brier score		0.14	0.14
*Forensic history*	*χ*^2^(4) = 3.64, *p* = 0.46		
Nagelkerke *R*^2^		< 0.01	< 0.01
Brier score		0.14	0.14
*Child maltreatment*	*χ*^2^(7) = 24.72, *p* < 0.001		
Nagelkerke *R*^2^		0.05	0.03
Brier score		0.14	0.14
*Social support*	*χ*^2^(2) = 1.18, *p* = 0.55		
Nagelkerke *R*^2^		< 0.01	< 0.01
Brier score		0.14	0.14
*Stigma*	*χ*^2^(1) = 0.01, *p* = 0.92		
Nagelkerke *R*^2^		< 0.01	< 0.01
Brier score		0.14	0.14
*Full model*	*χ*^2^(36) = 69.30, *p* = 0.001		
Nagelkerke *R*^2^		0.14	0.02
Brier score		0.13	0.14
Bias-corrected indexes have been bootstrapped (5000 replicates)

**Table 4:** Likelihood ratio tests comparing full model to domain-based models

	Likelihood ratio test
Demographic	*χ*^2^(31) = 60.24, *p* = 0.001
Financial security	*χ*^2^(34) = 68.78, *p* < 0.001
Mental disorder	*χ*^2^(24) = 38.24, *p* = 0.03
Substance use	*χ*^2^(34) = 68.50, *p* < 0.001
Functional impairment	*χ*^2^(35) = 69.29, *p* < 0.001
Forensic history	*χ*^2^(32) = 65.65, *p* < 0.001
Child maltreatment	*χ*^2^(29) = 44.57, *p* = 0.03
Social support	*χ*^2^(34) = 68.12, *p* < 0.001
Stigma	*χ*^2^(35) = 69.29, *p* < 0.001

The fully adjusted model was statistically significant, *χ*^2^(37) = 69.30, *p* = 0.001, but explained only 1.8% of the variance in CALD status (bias-corrected Nagelkerke *R*^2^ = 0.018). Two of the 31 variables entered in the model were significantly associated with CALD status. In addition to being less likely to report being diagnosed with a mental disorder during childhood, CALD participants reported higher levels of maternal overprotection. However, neither variable remained significant following correction (see supplementary information for raw and corrected *p*-values). Results did not differ substantially across datasets, suggesting they were largely unaffected by the presence of missing data (see supplementary information for full details).

## Discussion

There has been limited research on the mental health concerns of CALD Australians compared to majority culture populations. This study aimed to further our understanding by exploring differences in mental health problems and social factors between CALD and non-CALD young people presenting at a community based mental health services in metropolitan regions across the Australian states of Victoria and New South Wales. Similar levels of mental health symptoms, substance use, early adverse life experiences, and socioeconomic dynamics were identified cross-culturally. Although four correlates meaningfully differentiated between CALD and non-CALD populations, these findings were no longer significant after correcting for multiple testing. Comparisons across numerous clinical factors (e.g., general psychopathology, stigma, disability, clinical stage, quality of life, substance use, psychotic symptoms, depression/anxiety, criminal history) revealed no significant differences between groups.

Baseline characteristics were largely similar across groups. Recent clinical symptoms (for psychosis, anxiety, and depression) in particular, were comparable. Though limited, prior surveys have pointed to lower [11] and higher [12] levels of mental health concerns among non-English speaking migrants compared to Australian-born and overseas-born English speakers. Differences in findings likely reflect the nature of the surveys administered (i.e., clinical diagnostic criteria vs. generic symptoms). In our study, CALD status included Australian-born individuals with non-English speaking background ethnicities. This grouping perhaps encompassed many ‘second-generation’ individuals whose lifestyles may be consistent with those of the non-CALD group (for example, more than two-thirds of our CALD sample were born in Australia and several others born in English-speaking countries overseas). This may have engendered similarities in reporting, unlike prior research whereby ‘second generation’ CALD individuals are included in native-born populations [11, 12].

### Cultural considerations

A small number of cross-cultural differences were noted prior to multiple comparison adjustments. These bear some consideration and are potential avenues for further scientific inquiry. The CALD group was less likely to report a mental illness diagnosis during childhood, less likely to report a family history of mental illness or suicide, and presented with lower levels of sensation seeking and sensitivity to punishment/reward stimuli. Moreover, CALD participants were more likely to report maternal authoritarianism compared to non-CALD participants.

Lower reported levels of child-onset disorder were identified for CALD participants. The extent to which this finding reflects actual child prevalence rates across cultures in our sample, or rather the influence of sociocultural factors, is unknown. Community stigmas around mental illness are present within some CALD populations [22, 28, 29]. This may preclude families from seeking assistance from formal mental health service providers for mental health concerns. Evidence suggests that some CALD populations have a greater preference for informal support [23, 76]. Moreover, mental health literacy may be low or families may be unfamiliar with (or perhaps mistrust) mainstream mental health services [14, 22, 23, 77]. This may impede the possibility of an early childhood diagnosis for an affected family member with (in)attention to the behaviour, or remedies being handled ‘in house’. Some CALD youth may have spent the earlier parts of their childhood overseas where mental health services may be less established. Additionally, prior Australian research with CALD youth detected a minimisation of psychopathology [33], possibly the result of cultural shame or an unwillingness to share personal or humiliating experiences. This may induce differences between CALD and non-CALD participants when disclosing information on ‘child-onset disorder’ and ‘family history of mental illness or suicide’.

CALD youth presented with lower levels of drive, sensation seeking and sensitivity to punishment/reward stimuli. These items refer to self-reported involvement in fun-seeking behaviours and the aspiration of reward and the impulsive and/or persistent nature of this pursuit. No prior work has been conducted on how these traits may differ cross-culturally in Australia. Lower reported levels of these behaviours may reflect cultural differences in adolescent risk-taking. Western societies with ‘loose’ cultures (tolerant; relaxed social norms) are more likely to encourage exploration, assertiveness, permissiveness, and independence during adolescence [78, 79], whereas non-Western collectivist societies (particularly those with tight cultures) favour self-control, cooperation, cautiousness, and socially restrained behaviours [79–81]. CALD youth in our study may have possessed some of the latter traits. While the bulk of CALD youth were born in Australia, many may have been raised in bicultural environments where elements of their parents’ culture of origin were preserved. Maternal overprotection was associated with CALD status, as was maternal authoritarianism when analysing domain-specific models. Disciplinarian parenting styles in CALD communities have been noted in prior literature [82, 83]. While this style of parenting may restrict youth autonomy and subsequent risk-taking behaviour, it has also been found to incite familial intergenerational conflict and youth disconnection in acculturating migrant families [84, 85].

### Implications

Study findings indicate that in a mental health help-seeking youth population possessing similar social demographics, cultural differences (CALD and non-CALD) appear to be minimal. This is unsurprising given that CALD youth in this sample are likely to be ‘second generation’ and inhabit more mainstream lifestyles. The fact that they accessed a mainstream mental service supports this contention. For clinicians working in such venues, expectations of cultural difference typically anticipated of first generation migrants, could perhaps be tempered for second-generation individuals. Prior Australian work suggests that for second generation CALD youth, rapport development, building trust, and understanding their unique challenges (i.e., negotiating two cultures, perceptions of racism) may warrant more attention rather than a focus on cultural idiosyncrasies and behaviours expected of recently arrived migrants [86]. Nevertheless, some CALD young people may inherit particular non-Western cultural norms if raised in bicultural environments or if they spent some part of their earlier life in a non-English speaking country. This could mean that mental health services may not have been previously accessed due to cultural stigmas or low mental health literacy, rendering psychopathologies and family histories of illness formally unrecognised or unrecorded. Efforts should be made by clinical staff to ascertain the extent to which historical undiagnosed mental health concerns may have transpired. Several frameworks exist to assist clinicians in tactfully extracting this information from CALD patients in non-direct, culturally appropriate ways [87]. It has been noted in prior research that some CALD young people may be less emotionally forthcoming in clinical encounters due to cultural expectations of stoicism and the suppression of feelings [87].

### Limitations

This study has several limitations. While the proportion of CALD participants in this sample is representative of their proportion in the general population, it may not reflect the demographics and help-seeking behaviours of CALD youth generally. Many CALD youth will still keep mental health concerns private or will seek assistance initially from a family member or trusted community member. The study also precluded participants who were unable to speak English, which would have excluded recently arrived CALD migrants from the sample. It is possible that the psychometric properties of the instruments employed in the study may not generalise to non-Western populations, however, this may be inconsequential given the probably high acculturation levels of the CALD sample. Moreover, the number of questionnaires administered may have engendered a degree of survey fatigue, which may have contributed to the amount of missing data. Finally, the self-report nature of the questionnaires may have engendered under-reporting from CALD participants who may have minimised personal and familial behaviour deemed to be shameful or humiliating.

## Conclusion

This study explored differences in mental health problems and social factors between CALD and non-CALD young people presenting at a community based mental health service in Australia. Similar mental health and socioeconomic profiles were identified. A small number of correlates discriminated between CALD and non-CALD participants (mental illness diagnosis during childhood, family history of mental illness/suicide, sensation seeking, sensitivity to punishment, maternal overprotection) however these factors were no longer meaningful after adjustment for multiple comparisons. In help-seeking mainstream youth populations, cultural differences across clinical factors appear to be minimal.

## Data Availability

The data that support the findings of this study are available from author RP but restrictions apply to the availability of these data, which were used under license for the current study, and so are not publicly available.

## Abbreviations

ASSIST: The alcohol, smoking and substance involvement screening test
BIS/BAS: The behavioural inhibition/behavioural activation system
CAARMS: Comprehensive assessment of the at-risk mental state
CALD: Culturally and linguistically diverse
CGI: Clinical global impressions scale
CTQ: The childhood trauma questionnaire
DSM-IV: Diagnostic and statistical manual of mental disorders
FDR: False discovery rate
GAD-7: The generalized anxiety disorder scale
K-10: The Kessler 10
KMO: Kaiser–Meyer–Olkin
MD: Mean differences
MI: Multiple imputation
MICE: Multiple imputation by chained equations
OASIS: The overall anxiety severity and impairment scale
OR: Odds ratios
NEWQOL: Newly diagnosed epilepsy instrument
PBI: Parental bonding instrument
QIDS-C: Quick inventory of depressive symptomatology
PCA: Principal components analysis
RA: Research assistant
SOFAS: The social and occupational functioning scale
WHODAS: World Health Organization disability assessment schedule
WHOQOL: World Health Organization quality of life
YMRS: The young mania rating scale
